# Comprehensive Analysis of miRNA-Mediated Regulatory Network and Identification of Prognosis Biomarkers in Rectal Cancer

**DOI:** 10.3389/fgene.2022.792984

**Published:** 2022-04-12

**Authors:** Tingting Tang, Sisi Yu, Zekai Song, Xiaofu Pan, Fang Xu, Yanke Wu, Liang Zhang

**Affiliations:** ^1^ Department of Pathology, Ruian People’s Hospital, Zhejiang, China; ^2^ Department of Laboratory Medicine, Ruian People’s Hospital, Zhejiang, China; ^3^ Department of Digestive System, Ruian People’s Hospital, Zhejiang, China

**Keywords:** rectal cancer, prognosis markers, miRNAs, lncRNA, network

## Abstract

Rectal cancer is a malignant tumor with poor prognosis. Identification of prognostic biomarkers is needed to improve overall survival of rectal cancer patients. Here, we firstly identified *miR-20a-5p* significantly classifying high-risk group and low-risk group of rectal cancer patients. We also found that several known miRNAs *miR-142-5p*, *miR-486-5p*, *miR-490-3p* and *miR-133a-3p* played important roles in rectal cancer. Secondly, we constructed and analyzed a rectal cancer-related miRNA-mRNA network. A rectal cancer-related functional module was identified from the miRNA-mRNA network. Survival analysis demonstrated great prognosis capacity of the module to distinguish rectal cancer patients. Thirdly, a rectal cancer-related miRNA-lncRNA network was constructed, which followed power law distribution. Hub miRNAs and lncRNAs of the network were suggested to show significant prognosis ability and be enriched in cancer-related pathways. Fourthly, we constructed a rectal cancer-related ceRNA network and detected several typical lncRNA-miRNA-mRNA crosstalk, such as *HAND2-AS1*, *HAND2* and *miR-20a-5p* crosstalk and *MBNL1-AS1*, *miR-429* and *LONRF2* crosstalk, which were validated to function in improving overall survival of rectal cancer patients. Finally, we identified the regulatory feedback that was constituted by transcriptional factors and lncRNAs, including *MEIS1*, *MEIS2* and multiple lncRNAs. We also demonstrated that these lncRNAs were high related to immune cell infiltration. All these results can help us to uncover the molecular mechanism and provide new light on miRNA-mediated gene crosstalks in rectal cancer.

## Introduction

Rectal cancer is a kind of malignant tumor that happens on rectum, the risk factors of which are very widespread. The etiology of rectal cancer is not clear at present, may be related to environmental factors, dietary habits and genetic factors (Gaertner et al., 2015). The improved surgical techniques and the addition of neoadjuvant radiation therapy have been performed for patients of rectal cancer (Ludmir et al., 2017). But if the treatment of rectal cancer is not timely, local recurrence or distant metastasis may occur after the operation, causing serious complications. Eventually, cachexia leads to multiple organ dysfunction or failure and death (Li et al., 2016). The 5-years survival rate of patients with early rectal cancer is more than 90%, while that of patients with late rectal cancer is less than 50% (Dossa et al., 2018). It remains low in spite of the progress of diagnostic and therapeutic tools. Therefore, there is a critical need of biomarkers predicting pathological characteristics and subsequently improving patients’ prognosis.

The microRNAs (miRNAs), about 19-25 nt in length, are a class of endogenous, single-stranded, non-coding, small molecular RNAs (Lagos-Quintana et al., 2001; Lee et al., 2003). MiRNAs can control gene expression mainly by inhibiting translation or causing degradation of target RNAs (Hausser and Zavolan 2014). Because of the highly conserved roles of gene expression regulation, miRNAs may be of great interest as possible biomarkers for physiological processes (Vegter et al., 2016). Actually, miRNAs have been involved in various biological processes such as cell differentiation, cellular proliferation, metabolism, and apoptosis (Bartel 2004). The impact of specific miRNAs has been shown for almost every cancer. Long non-coding RNAs (lncRNAs) are a type of RNA transcripts that were once considered as transcriptional “noise” without protein-coding capacity, which are more than 200 nucleotides (Jathar et al., 2017). LncRNAs were reported to be closely involved in human diseases. For example, *HOTAIR*, *HULC*, and *linc00152* were reported to function in the occurrence and development of cancers, the high expression levels of which predicted a poor prognosis (Li et al., 2015; Xu et al., 2016). Zhang et al. suggested the lncRNAs *DBET, LINC00909, FLJ33534*, and *HSD52* were associated with neoadjuvant chemoradiotherapy (NCRT) response and prognosis in the rectal cancer (Zhang et al., 2021). For a given cell, the transcripts such as lncRNAs or mRNAs containing similar miRNA response elements (MREs) can regulate each other by competitively binding to common miRNAs, and thus act as miRNA sponge. This phenomenon is called competitive endogenous RNA (ceRNA) theory (Salmena et al., 2011). This competition plays crucial roles in tumorigenesis by affecting the expression levels of different kinds of RNAs. For example, *linc01133* regulated the expression of *APC* by sponging *miR-106a-3p* and further inhibited the progression of gastric cancer (Yang et al., 2018). Overexpression of lncRNA *HOXD-AS1* competitively bound to *miR-130a-3p* and prevented the degradation of *SOX4*, thus promoted the metastasis of hepatocellular carcinoma (Wang et al., 2017). Therefore, understanding this novel RNA crosstalk will have implications in human disease development. Meanwhile, transcriptional factors (TFs) interact with lncRNAs/miRNAs to regulate of cell cycle. LncRNA HAND2-AS1 was found significantly low in the rectal cancer tissues, which could interact with miR-1275 by target KLF14 to inhibit tumour (Cai et al., 2021).

All these genes including miRNAs, lncRNAs or mRNAs may play important roles in rectal cancer progression. However, genes usually do not function in isolation, they can interact with each other and be grouped into molecular networks. Thus, in the present study, we extracted rectal cancer-related miRNA/lncRNA/mRNA expression profile and constructed miRNA-mRNA network, miRNA-lncRNA network and miRNA-lncRNA-mRNA ceRNA network, respectively. In the context of these biomolecular networks, survival analysis was used for identifying novel biomarkers and functional modules associated with the diagnosis and prognosis of rectal cancer (Supplementary Figure S1).

## Materials and Methods

### Data Sets

We downloaded The Cancer Genome Atlas (TCGA) gene expression profile including transcript-level data of the same 89 tumor samples and three adjacent non-tumor samples from UCSC XENA browser (https://xenabrowser.net/datapages/). According to gene ID conversion from GENCODE (https://www.gencodegenes.org/human/), we converted transcripts with Ensembl IDs to lncRNAs and mRNAs with Gene Symbols. Similarly, we also converted transcripts to miRNAs based on ID conversion that supported by miRBase (https://www.mirbase.org/). In the process of ID conversion, if multiple transcripts corresponded to one miRNA/lncRNA/mRNA, mean expression value of multiple transcripts was computed as the expression value of the miRNA/lncRNA/mRNA. We performed log2 transformation for standardizing raw expression values and finally obtained miRNA expression profile, lncRNA expression profile and mRNA expression profile of rectal cancer with the same samples, respectively.

### Differential Expression Analysis

The edgeR test was used to calculate rectal cancer-related differentially expressed (DE) miRNAs, lncRNAs and mRNAs under the threshold of 2-fold change (FC) and *p*-value <0.05.

### Construction of Rectal Cancer-Related miRNA-mRNA Network

Firstly, the curated 423,975 miRNA-mRNA interactions between 386 miRNAs and 13,861 mRNAs were downloaded from starBase (Li et al., 2014). starBase is a comprehensive database which provided interaction networks of lncRNAs, miRNAs, ceRNAs and mRNAs from extensive CLIP-Seq (HITS-CLIP, PAR-CLIP, iCLIP, CLASH) data. Secondly, DE miRNAs and DE mRNAs were mapped into these interactions for extracting DE miRNA-DE mRNA interactions. Then, Pearson correlation coefficients (PCCs) between these DE miRNAs and DE mRNAs were computed based on miRNA expression profile and mRNA expression profile with the same samples. We only retained negatively expression-correlated DE miRNA-DE mRNA interaction pairs and constructed a rectal cancer-related miRNA-mRNA network.

### Construction of Rectal Cancer-Related miRNA-lncRNA Network

With the sequences of DE miRNA and DE lncRNA as input data, DE miRNA-DE lncRNA interactions were obtained using the miRanda tools (Enright et al., 2003) with default parameters. PCCs were calculated for these interaction pairs based on miRNA expression profile and lncRNA expression profile with the same samples. The DE miRNA-DE lncRNA pairs that expressed negatively correlated were extracted for constructing a rectal cancer-related miRNA-lncRNA network.

### Construction of Rectal Cancer-Related ceRNA Network

According to the above miRNA-mRNA network and miRNA-lncRNA network, we extracted lncRNA-mRNA pairs that shared at least one common miRNA. PCCs between these lncRNA-mRNA pairs were calculated based on lncRNA expression profile and mRNA expression profile of rectal cancer with the same samples. The lncRNA-mRNA pairs with PCC >0.9 were retained for constructing a rectal cancer-related ceRNA network.

### Construction of Rectal Cancer-Related TF-lncRNA Network

We obtained TFs list from AnimalTFDB (http://bioinfo.life.hust.edu.cn/AnimalTFDB/#!/) were mapped into the rectal cancer-related miRNA-mRNA network for screening TF-miRNA pairs. Previous research found that lncRNA could interacted with miRNA to regulate TF expression. Hypergeometric test was used for extracting TF-lncRNA pairs based on the number of common miRNAs between these TF-miRNA pairs and the above rectal cancer-related miRNA-lncRNA network. The heat map displayed the significant TF-lncRNA pairs with a threshold of *p*-value <0.05, containing 12 TFs and 17 lncRNAs (Figure 5A). To further identify the binding potential of TFs to lncRNAs, we defined the promoter region of a lncRNA as a basal domain of -2 kb to +2 kb around the transcriptional start site (TSS). We also downloaded enhancer regions from FANTOM5 project (Noguchi et al., 2017). A lncRNA was considered as the target of an enhancer if the enhancer located in more than ± 2 kb of the TSS of the lncRNA. Then, Find Individual Motif Occurrences (FIMO) (Izzat and Yim 1997) was used for performing motif occurrence (Grant et al., 2011). Results demonstrated that the motifs of TF MEIS1 and MEIS2 could bind to the promoters and enhancers of multiple lncRNAs with a threshold of FIMO *p*-value <1e–4, respectively (Figures 5B,C). These binding pairs also showed strong correlation.

### Analysis of Topological Features

Using the R package “igraph”, topological features such as network degree, cluster coefficient and average path length were calculated and analyzed for the network. Degree is the number of direct neighbors of nodes in the network. Cluster coefficient is the aggregation extent of nodes in the network graph. And average path length is the average value of the shortest paths between every two nodes of the network. To measure the statistical significance, we randomly produced 1,000 random networks with remaining the degree of nodes unchanged. The cluster coefficient and average path length were all calculated for the 1,000 random networks. The empirical *p*-values were respectively computed by the proportion of cluster coefficient in random network larger than that in the real network and the proportion of average path length in random network shorter than that in the real network.

### Identification of Functional Modules

MCODE can automatic prediction of protein complexes from qualitative protein-protein interaction data, so it can predict the function of unknown proteins and help understand the functional connections of molecular complexes in cells (Bader and Hogue 2003). Based on the miRNA-mRNA network, miRNA-lncRNA network and lncRNA-miRNA-mRNA ceRNA network, we used the Molecular Complex Detection (MCODE) plug-in in Cytoscape software to identify various rectal cancer-related functional modules (Shannon et al., 2003). The criteria of MCODE we used were as follows: MCODE scores >5, degree cut-off = 2, node score cut-off = 0.2, max depth = 100, and k-score = 2.

### Survival Analysis

For performing survival analysis, we downloaded clinical information of our rectal cancer samples from UCSC XENA. A risk model was constructed by calculating linear combination of the miRNA/lncRNA/mRNA expression values weighted by the regression coefficient of univariate Cox regression analysis. The following formula was used to calculate risk score:

$$RiskScore=\sum_{i=1}^{n}\beta_{i}Exp\left(i\right)$$

where, $\beta_{i}$ is the Cox regression coefficient of the *i*th miRNA/lncRNA/mRNA from an independent gene set; Exp(i) is the expression value of the *i*th miRNA/lncRNA/mRNA in a corresponding patient; and n is the number of miRNAs/lncRNAs/mRNAs in gene set.

The mean risk score was used as a cut-off to classify rectal cancer patients into high-risk group and low-risk group. A Kaplan-Meier survival curve was performed for different groups of rectal cancer patients. The statistical significance was assessed by log-rank test under the threshold of *p* < 0.05.

### Immune Cell Infiltration of lncRNAs in Patients

Cell infiltration information of rectal cancer patients were downloaded from TIMER2. The potential role of lncRNAs in cell infiltration was estimated by calculating the correlation between lncRNA expression and infiltration estimation scores.

## Results

### *miR-20a-5p* May be a Potential Prognosis Biomarker of Rectal Cancer

**FIGURE 1:**
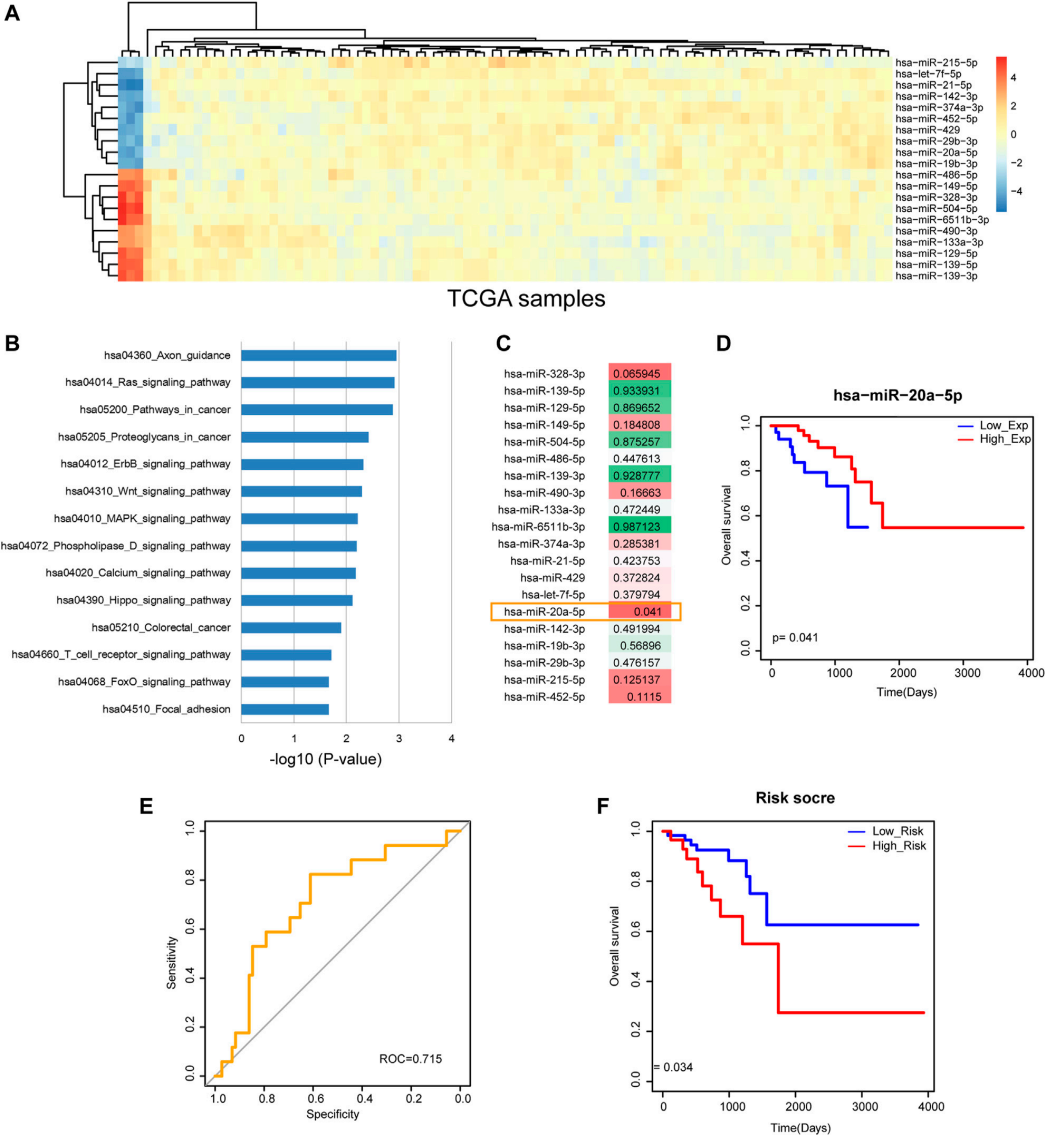
Identification of the potential prognosis biomarker of rectal cancer. **(A)** The heat map of the most upregulated 10 miRNAs and the most downregulated 10 miRNAs. **(B)** Pathway enrichment results of the 20 most DE miRNAs. **(C)** The log-rank *p*-values of the 20 most DE miRNAs. *miR-20a-5p* was statistically significant with *p* < 0.05. **(D)** A Kaplan-Meier survival curve of *miR-20a-5p* (*p* = 0.041). **(E)** ROC curve analysis result of the 20 most DE miRNAs. **(F)** A Kaplan-Meier survival curve of the 20 most DE miRNAs (*p* = 0.034).

From TCGA, we obtained rectal cancer-related miRNA expression profile containing 89 tumor samples and three adjacent non-tumor samples. The edgeR test with |FC| >2 and *p*-value <0.05 was used to identify rectal cancer-related DE miRNAs and totally 319 DE miRNAs were identified. Among the most upregulated 10 miRNAs and the most downregulated 10 miRNAs (Figure 1A), several miRNAs have been reported to function in rectal cancer. For example, *miR-215* was involved in response of rectal cancer to the chemoradiotherapy (Svoboda et al., 2012). ROC curve analysis showed that *miR-21* and *miR-328* could provide valuable information for individualizing treatment in rectal cancer patients (Campayo et al., 2018). 3D cell culture-based global miRNA expression analysis revealed increased levels of *miR-142-5p* in rectal tumor tissue samples after neoadjuvant long course treatment, which may be a theranostic biomarker of rectal cancer (Kunigenas et al., 2020). Low plasma level of exosomal *miR-486-5p* was associated with organ-invasive primary tumor, which attributed to adverse prognosis of rectal cancer (Bjornetro et al., 2019). The high diagnostic values of *miR-490-3p* and *miR-133a-3p* were shown in rectal cancer, which may provide a new way for treatment and prognosis improvement of digestive tract cancers (Lai et al., 2019). Pathway enrichment analysis was performed to the 20 most DE miRNAs that contained the most upregulated 10 miRNAs and the most downregulated 10 miRNAs. Results showed that they were enriched in some cancer-related pathways, such as “Pathways in cancer”, “ErbB signaling pathway”, “Wnt signaling pathway”, “MAPK signaling pathway” and “Focal adhesion” (Figure 1B). We further calculated risk score and the corresponding log-rank *p*-value for each of the 20 most DE miRNAs and found that *miR-20a-5p* was statistically significant (*p* < 0.05, Figure 1C). A Kaplan-Meier survival curve showed that *miR-20a-5p* significantly classified high-risk group and low-risk group of rectal cancer patients with different clinical outcomes (Figure 1D). It suggested that *miR-20a-5p* may be a potential prognosis biomarker of rectal cancer.

More interestingly, ROC curve analysis showed that the combination of the 20 most DE miRNAs had excellent capacity to distinguish rectal cancer patients with high-risk group from low-risk group (Figure 1E). And the Kaplan-Meier survival curve showed that the combination of the 20 most DE miRNAs significantly classified different risk groups of rectal cancer patients with different clinical outcomes (Figure 1F). These results demonstrated that the integrative analysis of miRNAs had significant prognosis capability, which deserved further research.

### MiRNA-mRNA Network and its Functional Module Show Prognosis Potential

**FIGURE 2:**
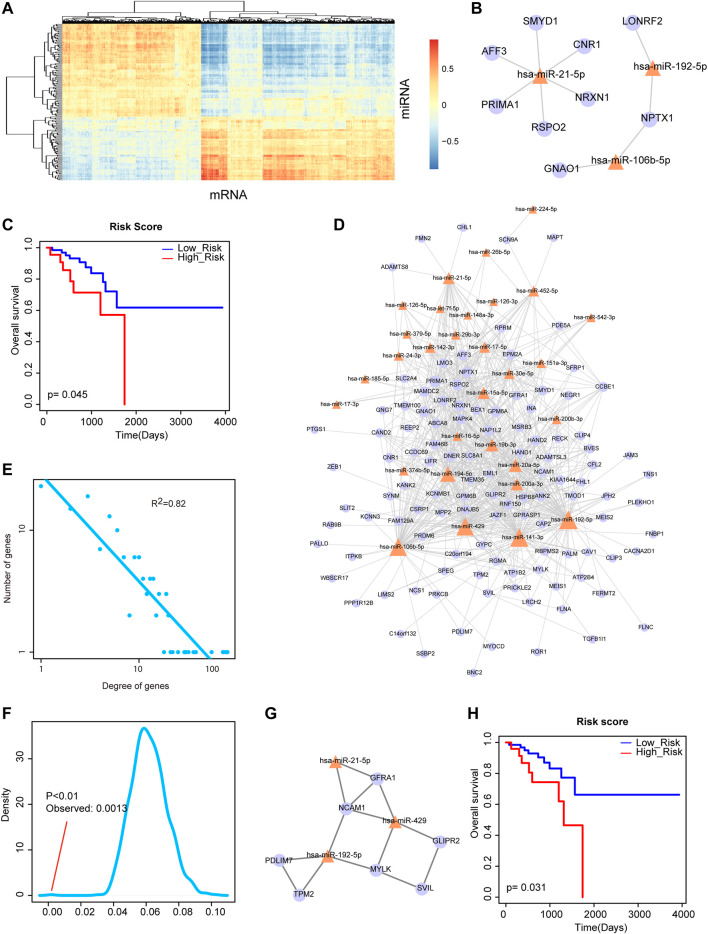
Analysis of miRNA-mRNA network and its functional module. **(A)** The heat map of correlations between DE miRNAs and DE mRNAs. **(B)** The most negatively expression-correlated 10 DE miRNA-DE mRNA interaction pairs. *miR-21-5p* was a hub node with the large degree. **(C)** A Kaplan-Meier survival curve of *miR-21-5p* and its six direct neighbors (*p* = 0.045). **(D)** The rectal cancer-related miRNA-mRNA network. Yellow triangle represents miRNA and purple circular represents mRNA. Node size represents degree of node. **(E)** Degree distribution of the network. All nodes follow a power-law distribution. **(F)** Average cluster coefficient of the real network was significantly larger than that of 1,000 random networks. **(G)** A functional module identified from the miRNA-mRNA network by MCODE. **(H)** A Kaplan-Meier survival curve of the three miRNAs of the module, *miR-21-5p*, *miR-429* and *miR-192-5p* (*p* = 0.031).

The rectal cancer-related DE miRNAs and DE mRNAs were mapped into the miRNA-mRNA interactions from starBase for extracting DE miRNA-DE mRNA interactions. As we all know, miRNAs negatively regulate the expression of target genes at the post-transcriptional level. Thus, we computed the correlations between these DE miRNAs and DE mRNAs by PCCs. The result of heat map showed that some DE miRNAs and DE mRNAs expressed negatively correlated (Figure 2A). We firstly extracted the most negatively expression-correlated 10 DE miRNA-DE mRNA interaction pairs and found that *miR-21-5p* was a hub node with the large degree (Figure 2B). Risk score was calculated for *miR-21-5p* and its six direct neighbors by linear combination of their expression values weighted by the regression coefficient of univariate Cox regression analysis. A Kaplan-Meier survival curve showed that they could significantly classify high-risk group and low-risk group of rectal cancer patients with log-rank *p* < 0.05 (Figure 2C). Secondly, we further detected the power of the combination of miRNAs and their target genes under the background of a larger network. Specifically, all the negatively expression-correlated DE miRNA-DE mRNA interaction pairs were used for constructing a rectal cancer-related miRNA-mRNA network, containing 702 interactions between 30 miRNAs and 114 mRNAs (Figure 2D). We calculated the degrees of all the nodes in the network. And the result of network degree distribution showed power law distribution (*R* ^2^ = 0.82, Figure 2E). We also computed cluster coefficients of the rectal cancer-related miRNA-mRNA network and 1,000 random networks generated by remaining the degree of nodes unchanged. Result showed that the average cluster coefficient of the real network was significantly larger than that of the random networks (*p* < 0.01, Figure 2F). Previous study suggested that the network with larger average cluster coefficient usually had modular structures. Therefore, we then identified a rectal cancer-related functional module from the miRNA-mRNA network by MCODE. The module was consisted of 3 DE miRNAs and 7 DE mRNAs (Figure 2G). Surprisingly, the three miRNAs of the module, including *miR-21-5p*, *miR-429* and *miR-192-5p* were the hub nodes of the rectal cancer-related miRNA-mRNA network. The result of survival analysis demonstrated their great capacity to distinguish rectal cancer patients with high-risk group from low-risk group (Figure 2H). These results suggested both the network and functional module that were consisted of DE miRNAs and their target DE mRNAs showed prognosis potential and played crucial roles in rectal cancer.

### MiRNA-lncRNA Network and Its Hub Nodes can Improve Overall Survival

**FIGURE 3:**
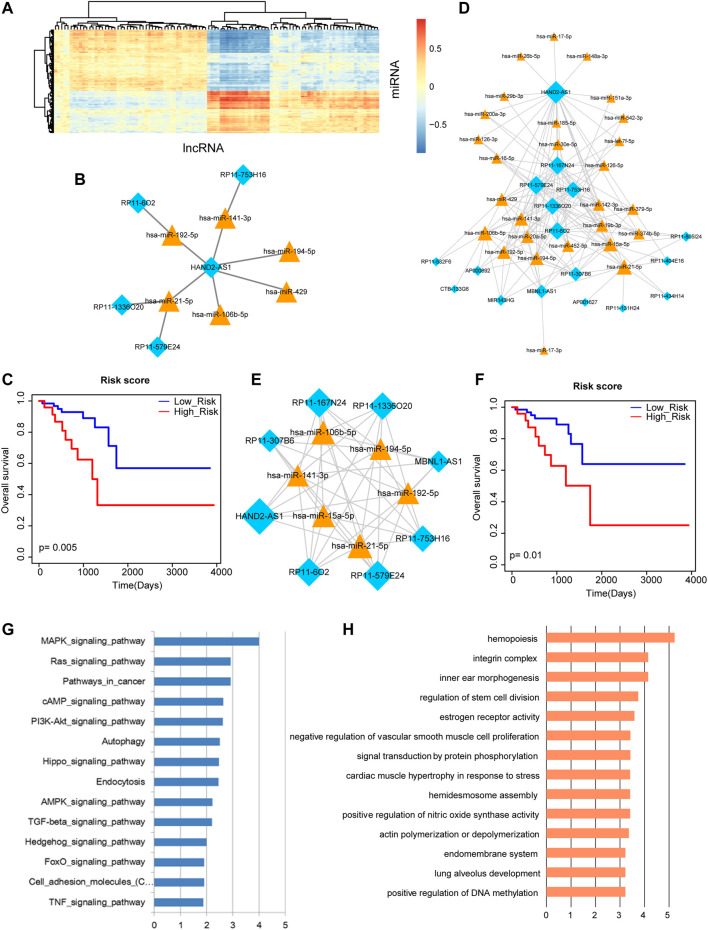
Analysis of miRNA-lncRNA network and its nodes. **(A)** The heat map of correlations between DE miRNAs and DE lncRNAs. **(B)** The most negatively expression-correlated 10 DE miRNA-DE lncRNA interaction pairs. **(C)** A Kaplan-Meier survival curve of the six miRNAs and five lncRNAs that express negatively correlated (*p* = 0.005). **(D)** The rectal cancer-related miRNA-lncRNA network. Yellow triangle represents miRNA and blue diamond represents lncRNA. Node size represents degree of node. **(E)** The top 20% miRNA and lncRNA hub nodes with the largest degrees in the miRNA-lncRNA network. **(F)** A Kaplan-Meier survival curve of these hub miRNAs and lncRNAs (*p* = 0.01). **(G)** Pathway enrichment analysis of these hub miRNAs. **(H)** GO Term enrichment analysis of these hub miRNAs.

By inputting the sequences of DE miRNAs and DE lncRNAs, DE miRNA-DE lncRNA interactions were obtained via the miRanda tools. The correlations between these DE miRNAs and DE lncRNAs were calculated by PCCs. The result of heat map showed that some DE miRNAs and DE lncRNAs expressed negatively correlated (Figure 3A). We extracted the most negatively expression-correlated 10 DE miRNA-DE lncRNA interaction pairs, referring to six miRNAs and five lncRNAs (Figure 3B). These miRNAs and lncRNAs were used for survival analysis. Risk score was calculated by linear combination of their expression values weighted by the regression coefficient of univariate Cox regression analysis. A Kaplan-Meier survival curve displayed their prognosis ability by distinguishing different risk groups of rectal cancer patients (Figure 3C). The result revealed that the combination of DE miRNAs and their target lncRNAs may contribute to the overall survival of rectal cancer patients. Therefore, we further conducted our analysis from the perspective of miRNA-lncRNA network. A rectal cancer-related miRNA-lncRNA network was constructed by integrating all the negatively expression-correlated DE miRNA-DE lncRNA interaction pairs, containing 155 interactions between 27 miRNAs and 17 lncRNAs (Figure 3D). The network followed power law distribution that most nodes had small degrees but a few nodes had very large degrees. We selected the top 20% miRNA and lncRNA hub nodes with the largest degrees from the miRNA-lncRNA network, which was consisted of six miRNAs, eight lncRNAs and their 47 interactions (Figure 3E). All the expression values of these miRNAs and lncRNAs were used for calculating risk score and their corresponding log-rank *p*-value. Kaplan-Meier survival curve revealed the significant prognosis ability of these hub miRNAs and lncRNAs (Figure 3F). Finally, pathway enrichment analysis was performed to these hub miRNAs. They were demonstrated to be enriched in multiple pathways associated with biological processes and molecular functions of cancers, including “MAPK signaling pathway”, “Pathways in cancer”, “Autophagy”, “Endocytosis”, “TGF-beta signaling pathway”, and “TNF signaling pathway” (Figure 3G).

### RNA Crosstalk has Implications in the Survival of Rectal Cancer Patients

**FIGURE 4:**
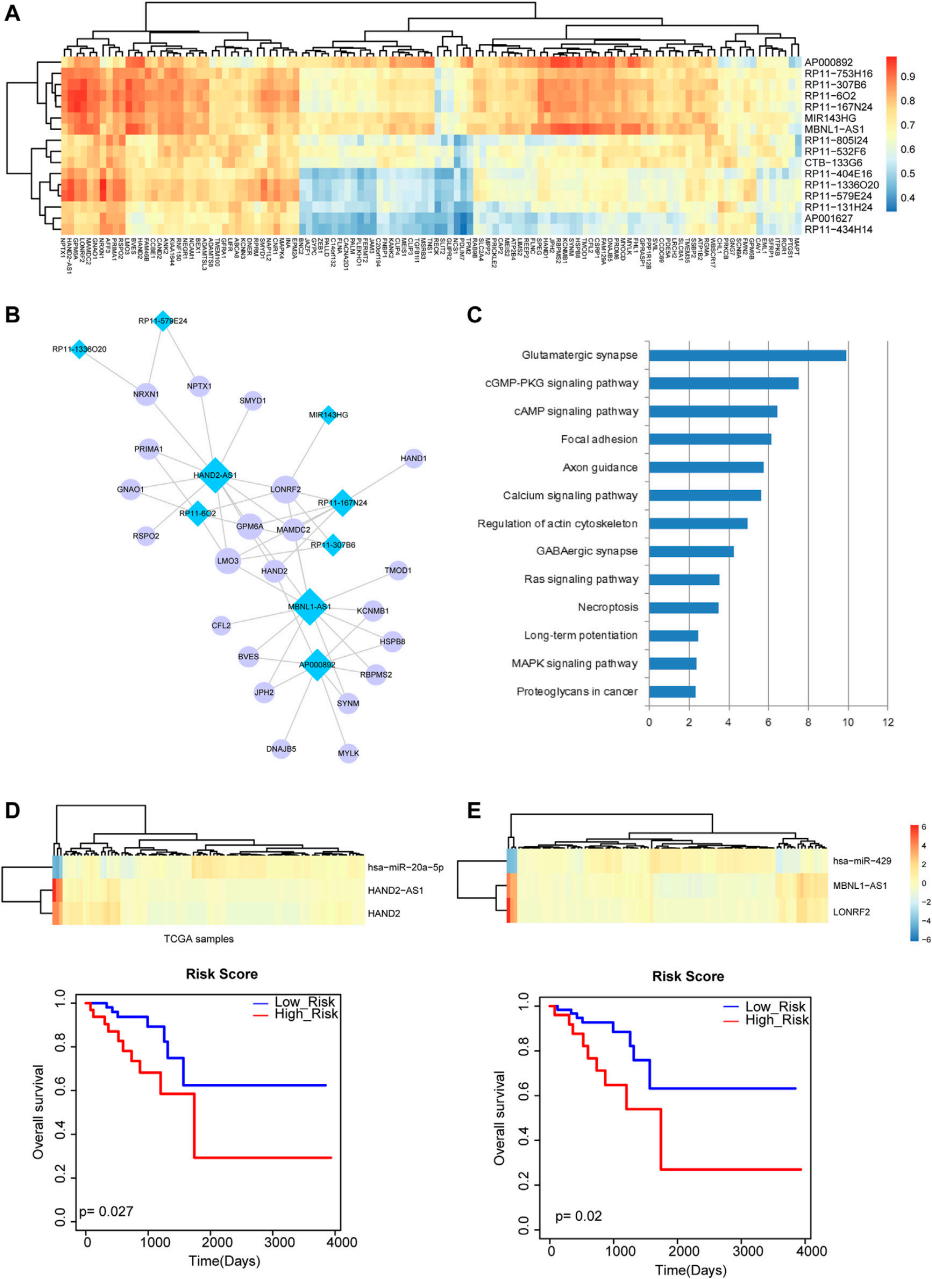
Analysis of lncRNA-mRNA network and lncRNA-miRNA-mRNA crosstalk. **(A)** The heat map of correlations between lncRNAs and mRNAs that shared at least one common miRNA. **(B)** The rectal cancer-related lncRNA-mRNA ceRNA network. Blue diamond represents lncRNA and purple circular represents mRNA. Node size represents degree of node. **(C)** Pathway enrichment analysis of the mRNAs in the network. **(D)** The heat map of *HAND2-AS1*, *HAND2* and *miR-20a-5p* and the Kaplan-Meier survival curve of the lncRNA-miRNA-mRNA crosstalk (*p* = 0.027). **(E)** The heat map of *MBNL1-AS1*, *miR-429* and *LONRF2* and the Kaplan-Meier survival curve of the lncRNA-miRNA-mRNA crosstalk (*p* = 0.02).

We computed correlations of lncRNAs and mRNAs that shared at least one common miRNA in the above miRNA-mRNA network and miRNA-lncRNA network by PCCs (Figure 4A). The lncRNA-mRNA pairs with PCC >0.9 were retained for constructing a rectal cancer-related ceRNA network, which referred to nine lncRNAs and 22 mRNAs (Figure 4B). The mRNAs of the network were used for pathway enrichment and were found to be related to multiple known cancer pathways, such as “Focal adhesion”, “Long-term potentiation” and “MAPK signaling pathway” (Figure 4C). In addition, in the rectal cancer-related ceRNA network, several lncRNAs were shown to have large degrees. In general, nodes with larger degrees are more important, which play vital roles in maintaining network integrity. Thus, we chose two lncRNAs *HAND2-AS1* and *MBNL1-AS1* with the largest degrees for in-depth analysis. According to lncRNA *HAND2-AS1* and its related mRNA *HAND2*, the miRNA *miR-20a-5p* that regulated them could also be extracted for detecting lncRNA-miRNA-mRNA crosstalk. Excitingly, *miR-20a-5p* was suggested to be a potential prognosis biomarker of rectal cancer in our previous analysis. We further performed survival analysis by computing linear combination of the expression values of *HAND2-AS1*, *HAND2* and *miR-20a-5p* weighted by the regression coefficient of univariate Cox regression analysis. A Kaplan-Meier survival curve represented prognosis ability of the lncRNA-miRNA-mRNA crosstalk (Figure 4D). Similarly, we identified another lncRNA-miRNA-mRNA crosstalk between *MBNL1-AS1*, *miR-429* and *LONRF2* and found that they could significantly distinguish high-risk group and low-risk group of rectal cancer patients (Figure 4E). These results suggested that the crosstalks between lncRNA, miRNA and mRNA may have implications in the survival of rectal cancer patients.

### TF *MEIS1* and *MEIS2* Coordinately Regulate Multiple lncRNAs

**FIGURE 5:**
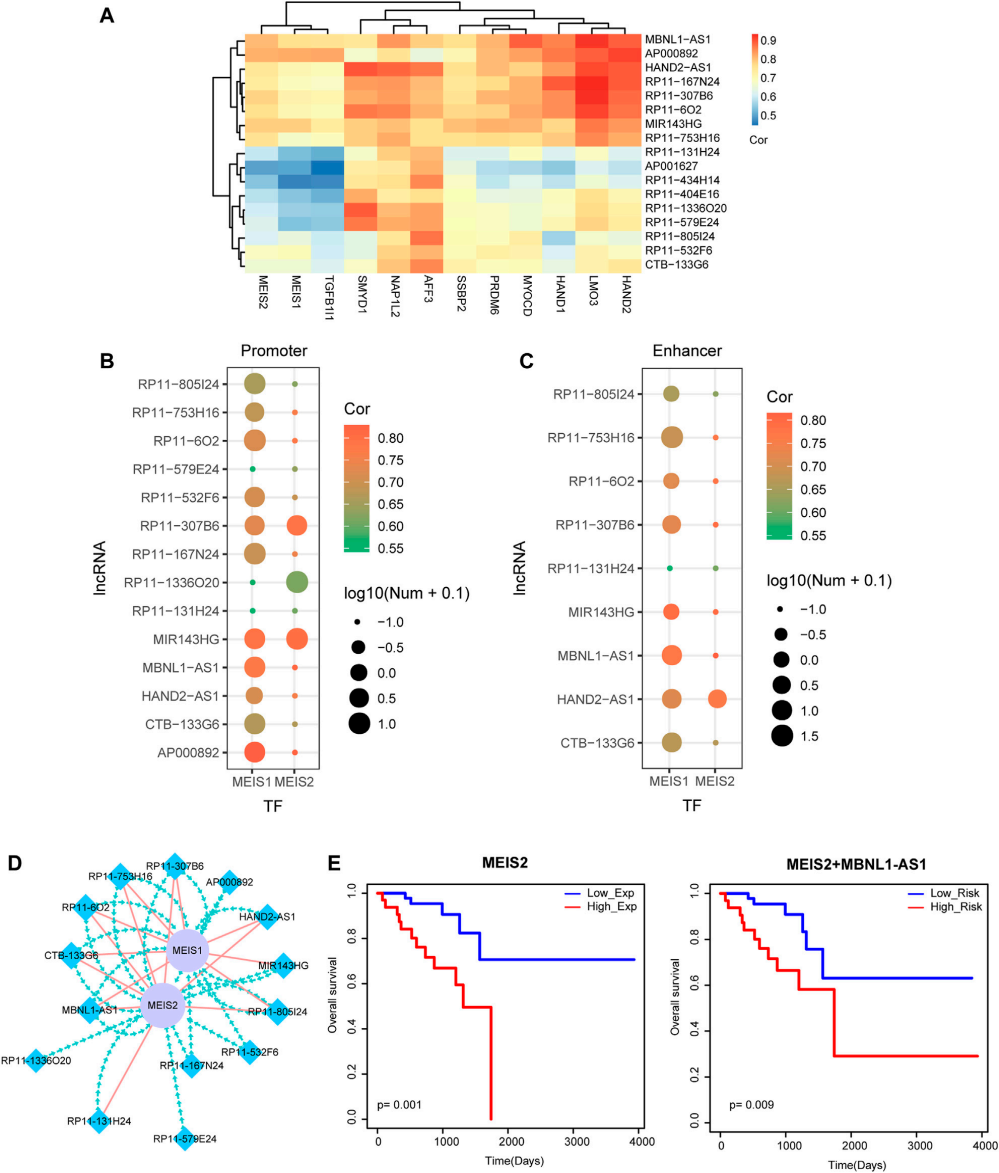
Identification of TF-lncRNA crosstalk based on motif analysis. **(A)** The heat map of TF-lncRNA pairs with a threshold of hypergeometric test *p*-value <0.05. **(B)** TF motif searching of promoter regions of lncRNAs. Node color represents the correlation score of PCC. Node size represents the number of TFs that bind to the promoter regions of lncRNAs. **(C)** TF motif searching of enhancer regions of lncRNAs. Node color represents the correlation score of PCC. Node size represents the number of TFs that bind to the enhancer regions of lncRNAs. **(D)** Visualization of a TF-lncRNA crosstalk network. Blue diamond nodes represent lncRNAs and purple circular nodes represent TFs. Green lines represent TFs binding to the promoter regions of lncRNAs. Red lines represent TFs binding to the enhancer regions of lncRNAs. **(E)** Kaplan-Meier survival curves of *MEIS2* and the combination of *MEIS2* and *MBNL1-AS1*.

Recently, studies have shown that TFs were involved in cancer pathology by regulating lncRNAs (Ji et al., 2020). We focused on the 2 TFs as well as the corresponding lncRNAs that had motif binding relationships and constructed a small TF-lncRNA crosstalk network (Figure 5D). We found that *MEIS1* and *MEIS2* coordinately regulated multiple lncRNAs by binding to their promoter or enhancer regions. For example, TF *MEIS1* and *MEIS2* simultaneously bound to the promoters and enhancers of lncRNA *MBNL1-AS1* and further coordinately regulated lncRNA *MBNL1-AS1*. Interestingly, lncRNA *MBNL1-AS1* has been demonstrated to be a hub node of the rectal cancer-related miRNA-lncRNA network. Finally, survival analysis was performed to *MEIS2* and the combination of *MEIS2* and *MBNL1-AS1*, respectively. The Kaplan-Meier survival curves displayed their prognosis ability by significantly distinguishing high-risk group and low-risk group of rectal cancer patients (Figure 5E).

**FIGURE 6:**
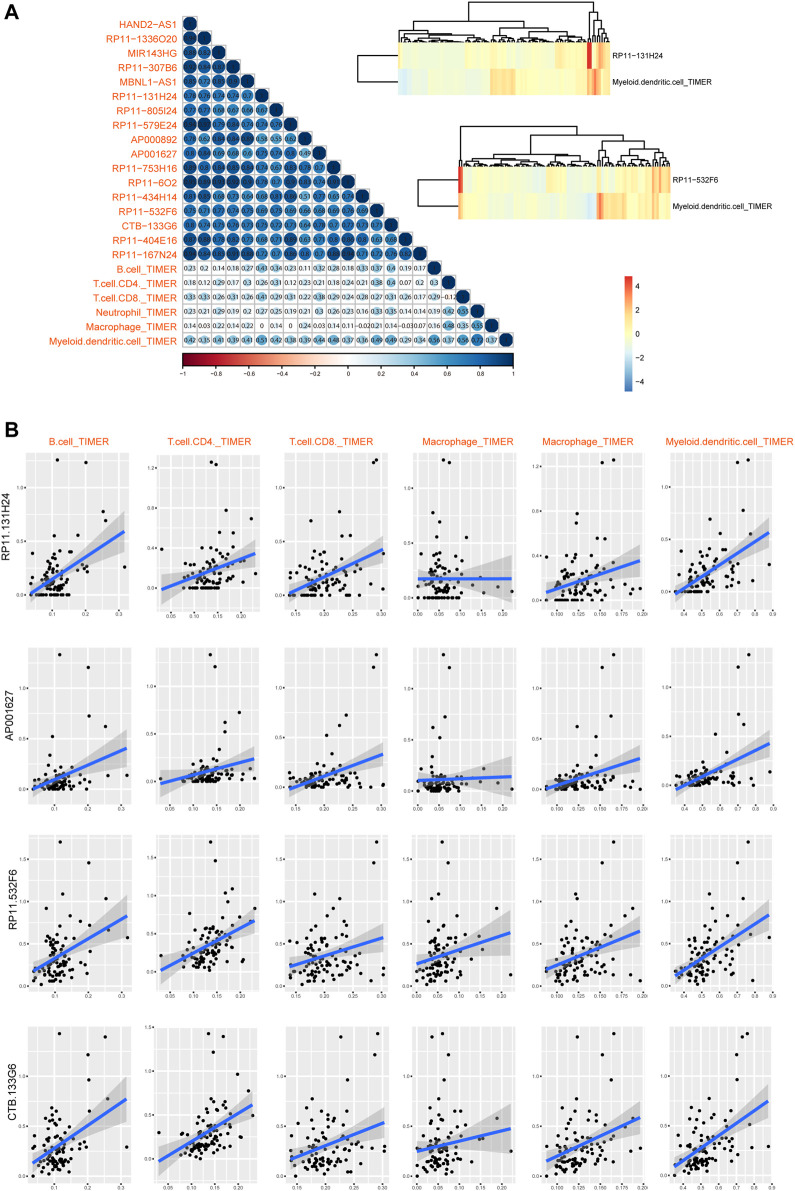
Immune cell infiltration of lncRNAs in READ patients. **(A)** The visualization of correlations between lncRNA expression and TIMER2 immune cell estimation score. Two highest correlation pairs were showed in heatmaps with Cor = 0.52 and 0.49. **(B)** Scatter plots of correlations between lncRNA expression and TIMER2 immune cell estimation score.

Furthermore, we also calculated the relationships between these lncRNAs and immune cell levels via integrating expression data and TIMER2 data. Results showed that myeloid dendritic cells were high related to these lncRNAs (Figure 6A). Especially, four lncRNAs were high related to immune cells than other transcripts, including RP11-131H24, AP001627, RP11-532F6 and CTB-133G6 (Figure 6B). These results also implied that lncRNAs might participate in cancer regulation by controlling immune cell levels in READ.

## Discussion

Rectal cancer is one of the most common cancers worldwide. More and more people suffer from rectal cancer due to the poor living and eating habits. The preoperative chemoradiotherapy and postoperative chemoradiotherapy have all been validated as effective treatments for patients of rectal cancer (Sauer et al., 2004). However, for patients with undifferentiated cancer, obvious regional lymph node metastasis or distant metastasis through the serous layer and surrounding infiltration, recurrence and metastasis are likely to occur 1–3 years after surgery and postoperative chemoradiotherapy, finally leading to death (Bosset et al., 2006). In this respect, potential prognostic biomarkers may play an increasing role in the study of rectal cancer. However, this is a lack of global lncRNA-associated crosstalks in rectal cancer. Thus, in our study, we aimed at finding predictive biomarkers that not only improve local control and reduce toxicity but also ameliorate overall survival of rectal cancer patients.

MiRNAs negatively regulate the expression of target genes at the post-transcriptional level. Evidence has suggested that miRNAs can represent almost all cellular and molecular functions, because about 60% of human mRNAs are regulated by miRNAs (Azizian et al., 2016). Thus, it is not unexpected that miRNAs are involved in diverse cellular processes, such as cell differentiation, proliferation and apoptosis (Bartel 2009). In addition, abnormal expression of the miRNAs can lead to cell dysfunction and then result in the occurrence and development of various diseases, even cancer (Mestdagh et al., 2009; Slaby et al., 2009). Therefore, we focused on miRNAs’ function in rectal cancer and explored their potential to serve as possible prognosis biomarkers and novel therapeutic targets. In recent years, there have been some DE miRNAs identified in the progression of rectal cancer, though limited data on miRNAs in rectal cancer are available (Zhang et al., 2018; Zhou et al., 2018). In this study, a network-based computational analysis was performed to investigate the key lncRNAs and TFs in rectal cancer. We constructed rectal-related global lncRNA-TF network by integrating information of differentially expressed lncRNAs/TFs and then identified functional modules. We identified several known miRNAs that functioned in rectal cancer, such as *miR-142-5p*, *miR-486-5p*, *miR-490-3p* and *miR-133a-3p*. Meanwhile, we also found *miR-20a-5p* significantly classifying high-risk group and low-risk group of rectal cancer patients, suggesting that *miR-20a-5p* may be a potential prognosis biomarker of rectal cancer. These results demonstrated good topological features and integrative power of miRNAs and their target mRNAs/lncRNAs. The closely connected modules were shown to function in survival state of rectal cancer patients and have potential prognosis ability.

Among the abundant knowledge about miRNAs’ function and mechanism, ceRNA theory has been reported to play a vital regulatory role in almost every cancer. Most ceRNAs have potential MREs, share common miRNAs and compete for binding common RNAs. In this study, we constructed a rectal cancer-related ceRNA network and detected several typical lncRNA-miRNA-mRNA crosstalk, such as *HAND2-AS1*, *HAND2* and *miR-20a-5p* crosstalk and *MBNL1-AS1*, *miR-429* and *LONRF2* crosstalk, which were validated to play important roles in improving overall survival of rectal cancer patients. Importantly, we also identified a TF-lncRNA feedback loops based on ceRNA mechanism and motif analysis. Previous studies have proved that TF-lncRNA feedback could help us to uncover the molecular mechanism (Hong et al., 2020) (Swarr et al., 2019). We found that some TFs, such as *MEIS1* and *MEIS2* might function as the key regulators of lncRNAs in READ. TF-lncRNA pairs could also be used as prognosis markers. Furthermore, we found the TF-regulated lncRNAs were high related to immune cell levels.

Though novel biomarkers and functional modules associated with the diagnosis and prognosis of rectal cancer have been achieved in this study, there were still some limitations in our study. Firstly, limited data on miRNAs and lncRNAs in rectal cancer are available. We only used TCGA data in the study. If a large number of miRNA and lncRNA expression profile data are released, we may discover more valuable information. Secondly, the questions about survival, recurrence and metastasis of rectal cancer are extremely complicated. Survival analysis is just one way to measure the prognosis of rectal cancer from bioinformatics. If combined with the experimental research, we will understand the pathogenesis and molecular mechanism in depth.

## Data Availability

Publicly available datasets were analyzed in this study. This data can be found here: https://xenabrowser.net/datapages/.

## Supplementary Material

Supplementary Figure S1 The analysis protocol to identify miRNA-mediated prognosis markers in READ.
