# Supplementary figures and images for: Comprehensive Analysis of miRNA-Mediated Regulatory Network and Identification of Prognosis Biomarkers in Rectal Cancer
Source: Front Genet. 2022 Apr 12;13:792984. doi: 10.3389/fgene.2022.792984 (PMC9039402; doi:10.3389/fgene.2022.792984)

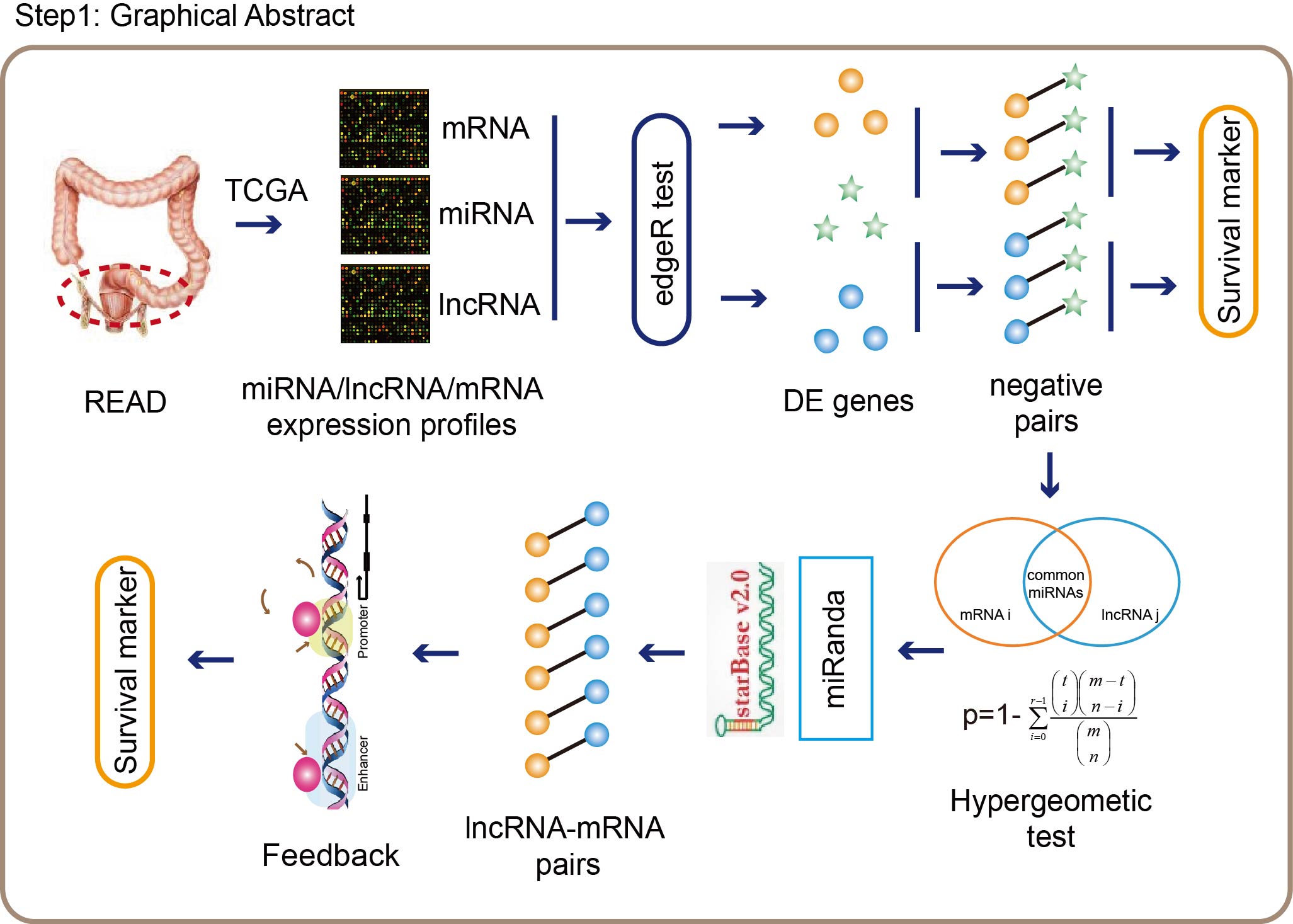

Supplement: Supplementary file 1 [file Image1.JPEG]
